# Clinical impact of normal alanine aminotransferase on direct‐acting antiviral outcome in patients with chronic hepatitis C virus infection

**DOI:** 10.1002/jgh3.12296

**Published:** 2019-12-23

**Authors:** Satoru Joshita, Ayumi Sugiura, Takeji Umemura, Tomoo Yamazaki, Naoyuki Fujimori, Akihiro Matsumoto, Yoko Usami, Eiji Tanaka

**Affiliations:** ^1^ Department of Medicine, Division of Gastroenterology and Hepatology Shinshu University School of Medicine Matsumoto Japan; ^2^ Department of Life Innovation, Institute for Biomedical Sciences Shinshu University Matsumoto Japan; ^3^ Consultation Centers for Hepatic Diseases Shinshu University Hospital Matsumoto Japan; ^4^ Department of Laboratory Medicine Shinshu University Hospital Matsumoto Japan

**Keywords:** direct‐acting antiviral, hepatitis C virus carrier with normal alanine aminotransferase, hepatitis C virus, hepatocellular carcinoma

## Abstract

**Background and Aims:**

This study aimed to clarify the clinical picture of hepatitis C virus (HCV) carriers with normal alanine aminotransferase (CNALT) and those with ALT elevation (non‐CNALT) under direct‐acting antivirals (DAAs).

**Methods:**

We enrolled 1002 patients with HCV (427 men, median age: 69 years) who had received DAAs for comparisons between CNALT (ALT ≤33 U/L in males and ≤25 U/L in females; *n* = 374) and non‐CNALT (*n* = 628) groups.

**Results:**

CNALT patients displayed a higher platelet count (PLT) (170 000 *vs* 146 000/μL, *P* < 0.0001) and albumin (4.1 *vs* 4.1 g/dL, *P* = 0.0006) but lower AST (25 *vs* 51 U/L, *P* < 0.0001), alpha fetoprotein (3.2 *vs* 5.4 ng/mL, *P* < 0.0001), and liver fibrosis marker scores (all *P* < 0.0001). The sustained virologic response rate was comparable between the CNALT and non‐CNALT groups (97.8 *vs* 95.3%, *P* = 0.106). The cumulative incidence of hepatocellular carcinoma (HCC) after DAA treatment was comparable between the CNALT and non‐CNALT groups (*P* = 0.117, log‐rank test). In CNALT patients with HCC history, PLT ≥150 000/μL was an independent risk factor of HCC recurrence (*P* = 0.019). In non‐CNALT patients without HCC history, male gender (*P* = 0.021) and albumin <4.0 g/dL (*P* = 0.007) were independent risk factors, while PLT < 150 000/μL (*P* = 0.081) was a marginal risk factor of HCC occurrence.

**Conclusion:**

CNALT patients displayed a milder degree of liver fibrosis. Combinations of CNALT and PLT status might be useful as markers for HCC occurrence or recurrence surveillance.

## Graphical abstract

Hepatitis C virus carriers with normal alanine aminotransferase displayed milder disease progression under modern direct‐acting antivirals era. Combinations of alanine aminotransferase and platelet count levels might be useful as markers for hepatocellular carcinoma occurrence or recurrence surveillance after direct‐acting antivirals.

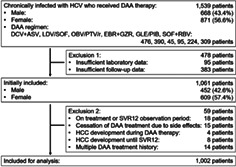

## Introduction

Chronic long‐term hepatitis C virus (HCV) infection eventually results in severe liver disease that includes advanced fibrosis, cirrhosis, and hepatocellular carcinoma (HCC).^1^ HCV eradication is the most effective treatment to halt disease progression. The development of direct‐acting antivirals (DAAs), which target specific steps within the HCV lifecycle,^2^, ^3^ has revolutionized therapy for HCV by providing high sustained viral response (SVR) rates of over 90%.^4^ However, issues such as HCC development and lipid profile changes after DAA treatment remain.^5^, ^6^, ^7^

The majority of patients with chronic HCV have mild, asymptomatic elevations in serum transaminase levels, with roughly a quarter exhibiting persistently normal alanine aminotransferase (ALT).^8^ As the degree of liver fibrosis in normal ALT HCV patients (CNALT) was considered minimal, such individuals were initially monitored conservatively without treatment. However, it was later realized in the interferon (IFN) era that a considerable number of CNALT patients displayed significant inflammation and fibrosis over time.^8^ The current clinical impact of CNALT on DAA outcome remains unknown. This study aimed to clarify the clinical features of CNALT and abnormal ALT HCV patients (non‐CNALT) receiving DAAs and identify the predictive factors of HCC development in these groups after DAA treatment.

## Methods

### *Patients*

**Figure 1:**
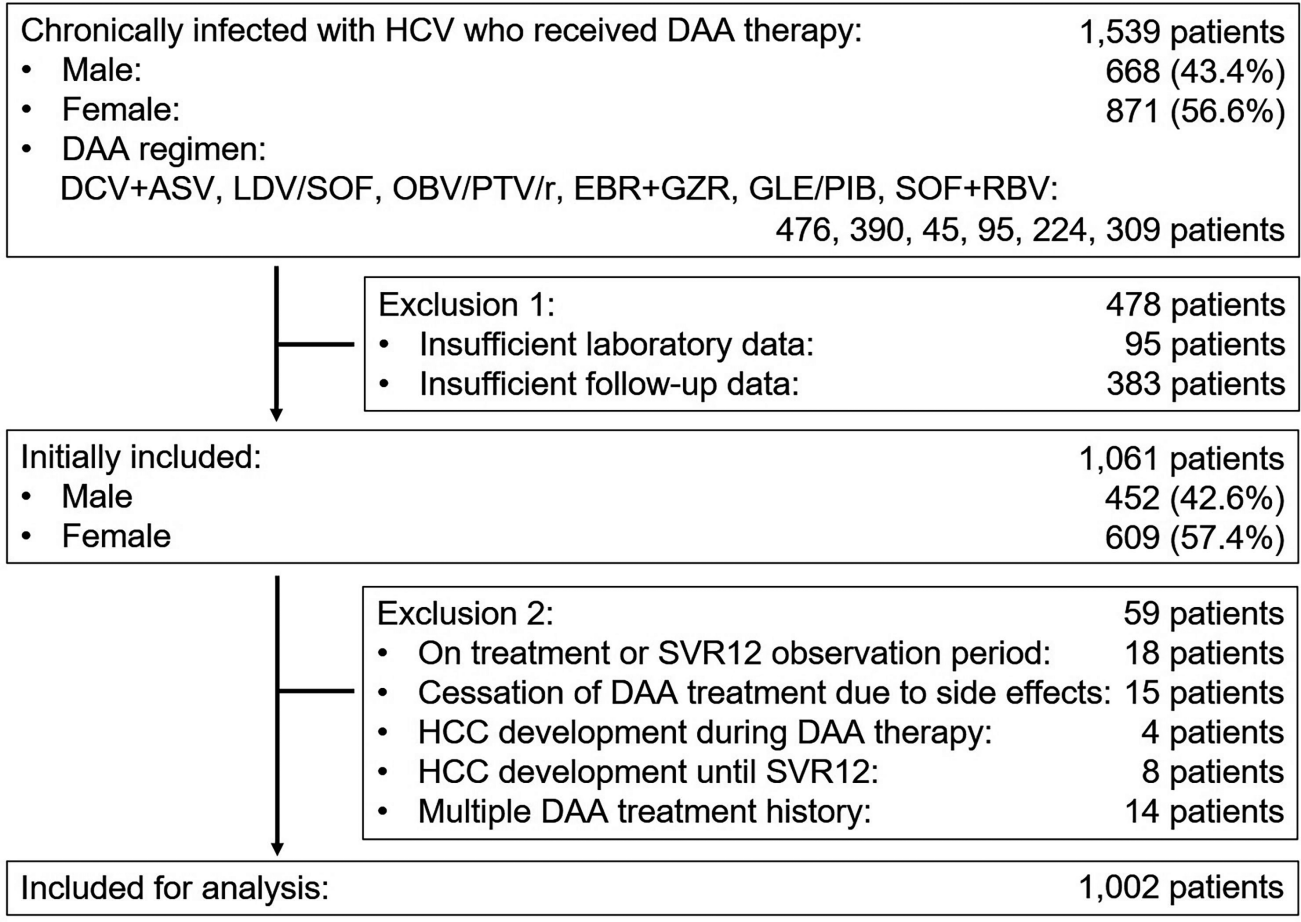
Patient selection in this study.

A total of 1539 patients chronically infected with HCV who received DAA therapy at Shinshu University Hospital (Matsumoto, Japan) or its affiliated hospitals between April 2015 and November 2018 were initially considered for this retrospective study. The number of patients who received daclatasvir+asunaprevir (DCV + ASV), ledipasvir/sofosbuvir (LDV/SOF), ombitasvir/paritaprevir/ritonavir (OBV/PTV/r), elbasvir+grazoprevir (EBR + GZR), glecaprevir/pibrentasvir (GLE/PIB), and sofosbuvir+ribavirin (SOF + RBV) were 476, 390, 45, 95, 224, and 309, respectively. Of them, 95 patients were excluded due to insufficient laboratory data, and 383 patients were removed due to insufficient follow‐up data. Of the remaining 1061 patients, 18 patients who were still receiving treatment or were in the SVR12 observation period, 15 patients with treatment cessation due to side effects, 4 patients experiencing HCC during DAA treatment, and 8 patients displaying HCC after DAA treatment during the SVR12 period were also excluded. An additional 14 patients with multiple DAA treatment history (2 with triple DAA treatment history) were dropped. Ultimately, 1002 patients were included for clinical comparisons between CNALT and non‐CNALT groups in this multicenter cohort analysis across Nagano Prefecture, Japan (Fig. 1). The median interval between SVR12 and the most recent outpatient clinic visit or HCC complication was 25.6 months (interquartile range: 68.6–32.5 months). The racial background of all subjects was Japanese. The diagnosis of chronic hepatitis C was based on the presence of serum HCV antibodies and detectable HCV RNA according to guidelines from the Japan Society of Hepatology.^9^

The study was approved by the Institutional Review Board of Shinshu University School of Medicine (approval number: 3244) and its affiliated hospitals. Written informed consent was obtained from all participating subjects. The study was conducted according to the principals of the Declaration of Helsinki.

### *Study design*

All patients were registered upon commencement of DAA treatment and treated with DAA regimens that included DCV + ASV for 24 weeks; LDV/SOF, OBV/PTV/r, or EBR + GZR for 12 weeks; GLE/PIB for 8 weeks in chronic stage patients or 12 weeks in cirrhosis stage patients, and SOF + RBV for 12 weeks according to guidelines from the Japan Society of Hepatology.^9^ We collected clinical information at the end of December 2018. An SVR was defined as undetectable HCV RNA at 12 weeks after the completion of DAA therapy (i.e. SVR12). Treatment failure was defined as detectable HCV RNA during DAA treatment or within 12 weeks of completion or discontinuation of DAAs. HCC was diagnosed by imaging studies. The HCC surveillance interval duration after DAA therapy differed in each case and depended on the attending physician.

### *Laboratory testing*

**Figure 2:**
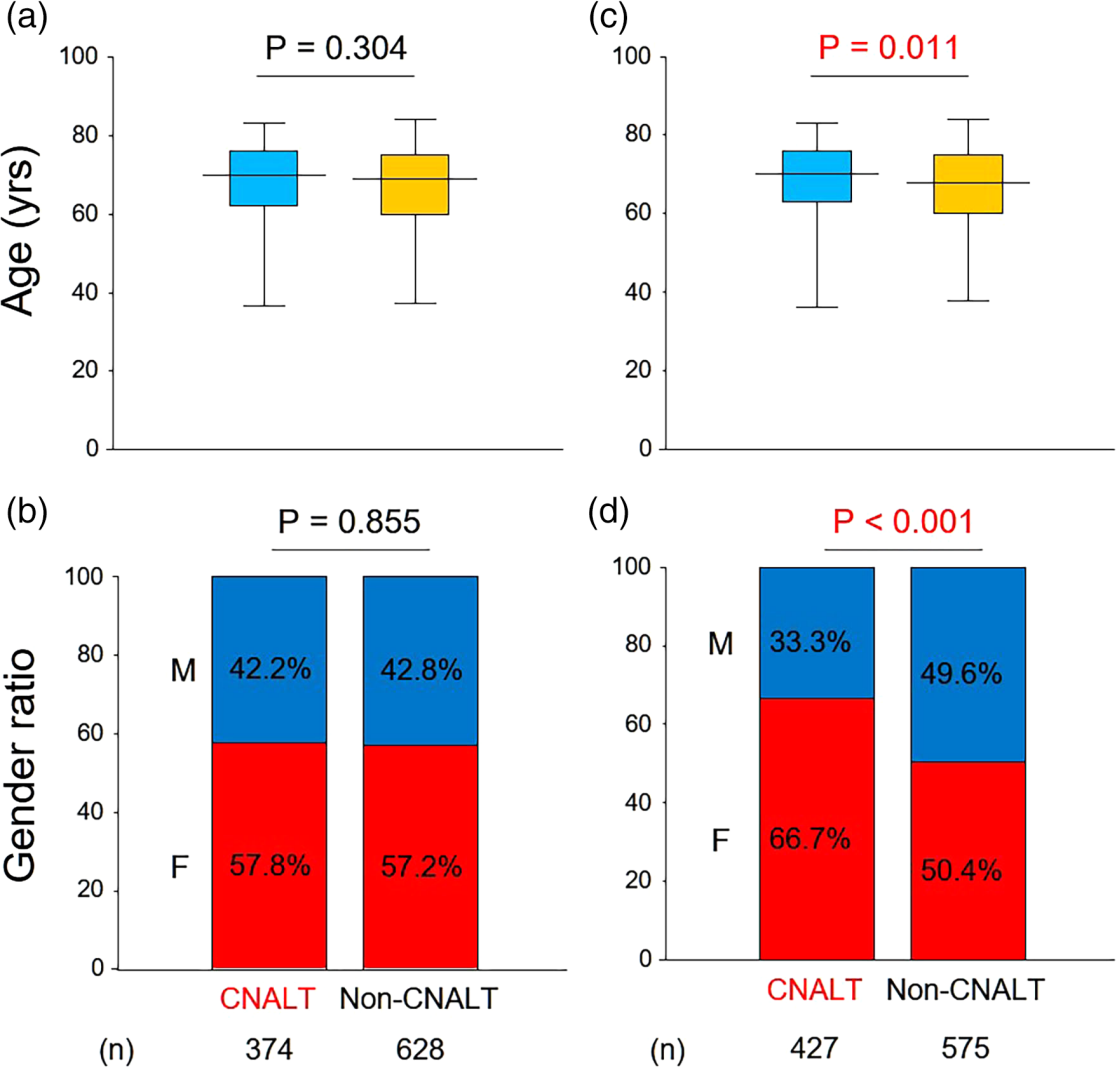
Age and gender differences between CNALT and non‐CNALT groups by two CNALT definitions. There were no significant differences for age (a) or gender ratio (b) between CNALT and non‐CNALT groups by considering physiological ALT differences between genders with ALT ≤33 U/L in males and ≤ 25 U/L in females, which became the thresholds adopted in this study. There were significant differences for age (c) and gender ratio (d) between CNALT and non‐CNALT groups by setting ALT at ≤30 U/L. CNALT, hepatitis C patients with normal alanine aminotransferase; F, female; M, male.

All laboratory data, including platelet count (PLT), albumin, aspartate aminotransferase (AST), alanine aminotransferase (ALT), and alpha fetoprotein (AFP), were determined using standard methods at respective institutions. When deciding on the CNALT threshold at DAA commencement, the simple establishment of ALT ≤30 U/L, as reported previously,^8^ produced significant differences in age and gender ratio between the groups (Fig. 2c,d). However, by considering the physiological ALT differences between genders, such differences disappeared with the establishment of ALT ≤33 U/L in males and ≤ 25 U/L in females, as described previously.^10^ This definition was adopted in the present study (Fig. 2a,b). Fibrosis markers, such as the FIB‐4 index^11^ and aspartate aminotransferase‐to‐platelet ratio index,^12^, ^13^ were respectively calculated, while macrophage galactose‐specific lectin‐2 binding protein glycosylation isomer (M2BPGi), which was quantified based on a lectin–antibody sandwich immunoassay using an HISCL‐5000 fully automatic immunoanalyzer (Sysmex Co., Hyogo, Japan), and autotaxin, which was quantified by a specific two‐site enzyme immunoassay using the AIA system (Tosoh Co., Tokyo, Japan), were measured using cryogenically stored serum samples, as described earlier.^14^, ^15^, ^16^, ^17^

### *Statistical analysis*

Statistical analysis was carried out using StatFlex ver. 7.0.2 software (Artech Co., Ltd., Osaka, Japan). The Mann–Whitney *U* and chi‐square tests were used where appropriate. Cumulative incidences of HCC after DAA therapy were compared by the Kaplan–Meier method with log‐rank test. A multivariate analysis was performed using Cox proportional regression analysis with stepwise method after categorizing continuous variables to minimize interference. All statistical tests were two‐sided and evaluated at the 0.05 level of significance.

## Results

### *Baseline clinical characteristics*

Of the 1002 enrolled patients with a median age of 69 years, 427 (42.6%) were male. The number of patients infected with genotypes 1 and 2 and other, including genotype 3, was 703, 295, and 4, respectively. Seventy patients (9%) were complicated with HCC that was already treated successfully and had no active tumors according to imaging findings just before DAA treatment. Therefore, no patient was considered to have active HCC at the commencement of DAAs, especially because DAA treatment has not been approved in Japan for active HCC. The overall SVR rate was 97.3% in the cohort (Table 1). The cumulative incidence of HCC after DAA treatment at 6 months, 12 months, and 24 months was 2.3, 3.1, and 6.4%, respectively. The cumulative incidence of HCC after DAA treatment was comparable between the genotype 1 and genotype 2 groups as analyzed by Kaplan–Meier analysis (*P* = 0.342, log‐rank test).

**Table 1:** Baseline characteristics and comparisons of clinical features between CNALT and non‐CNALT groups

	All patients (*n* = 1002)	IQR	CNALT (*n* = 374)	Non‐CNALT (*n* = 628)	*P* value
Age at entry (years)	69	60–75	70	69	0.306
Male, *n* (%)	427 (42.6)		158 (42.2)	269 (42.8)	0.855
Genotype 1/2/other	703/295/4		246/128/0	457/167/4	0.013
History of HCC, *n* (%)	70 (7.0)		23 (6.1)	48 (7.5)	0.423
Laboratory data					
Platelet count (/μL)	155 500	113 500–195 000	170 000	146 000	<0.0001
Platelet count <150 000/μL, %	45.5		35.5	51.3	<0.0001
Albumin (g/dL)	4.1	3.8–4.3	4.1	4.1	0.0006
AST (U/L)	37	27–57	25	51	<0.0001
ALT (U/L)	35	23–59	21	50	ND
AFP (ng/mL)	4.2	2.8–7.3	3.2	5.4	<0.0001
Fibrosis markers					
FIB‐4 index	2.95	1.91–4.67	2.34	3.48	<0.0001
APRI	0.64	0.37–1.67	0.37	0.89	<0.0001
M2BPGi (COI)	1.82	1.11–3.55	1.27	2.37	<0.0001
Autotaxin (mg/L)	1.50	1.11–2.05	1.26	1.63	<0.0001
DAA outcome of SVR, *n* (%)	975 (97.3)		366 (97.8)	609 (95.3)	0.106
Follow‐up period† (months)	25.6	8.6–32.5	23.8	26.2	0.040

† Interval between SVR12 and last outpatient clinic visit or HCC complication.

AFP, alpha fetoprotein; ALT, alanine aminotransferase; APRI, aspartate aminotransferase‐to‐platelet ratio index; AST, aspartate aminotransferase; CNALT, hepatitis C patients with normal alanine aminotransferase; COI, cutoff index; DAA, direct‐acting antiviral; FIB‐4, fibrosis‐4 index; HCC, hepatocellular carcinoma; IQR, interquartile range; M2BPGi, macrophage galactose‐specific lectin‐2 binding protein glycosylation isomer; ND, not determined; SVR, sustained virologic response.

### *Comparisons of clinical characteristics between CNALT and non‐CNALT*

**Figure 3:**
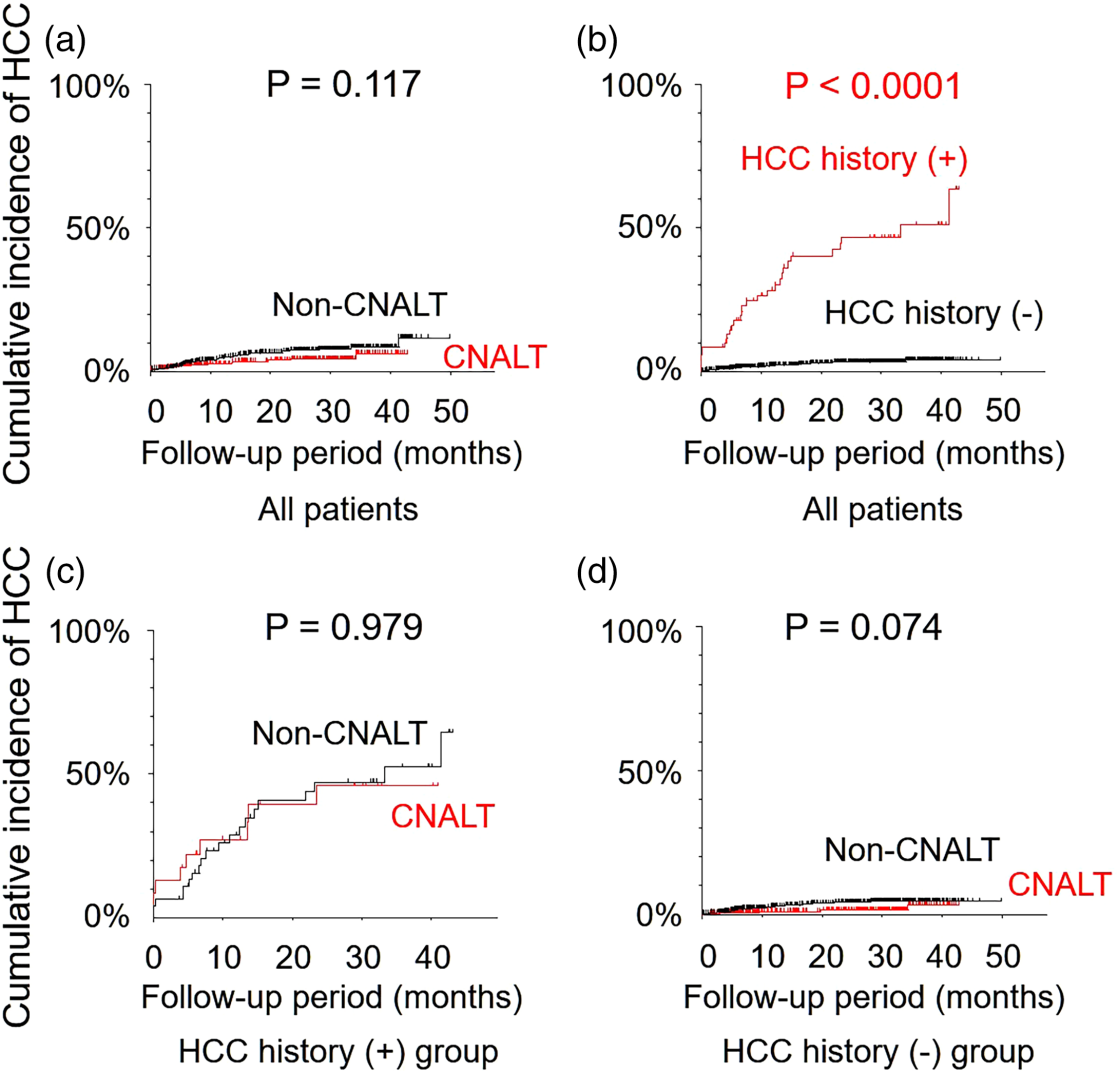
Cumulative incidence of HCC development after DAA treatment. There was no significant difference in the cumulative incidence of HCC development between CNALT and non‐CNALT groups (a). The HCC history (+) group showed a significantly higher cumulative incidence of HCC development than did the HCC history (−) group (b). There were no significant differences in HCC recurrence rate for the HCC history (+) group (c) or HCC occurrence for the HCC history (−) group (d) between CNALT and non‐CNALT patients. CNALT, hepatitis C patients with normal alanine aminotransferase; HCC, hepatocellular carcinoma.

Clinical characteristic comparisons between the CNALT and non‐CNALT patients are summarized in Table 1. PLT and albumin were significantly higher in the CNALT group, while AST and AFP were significantly lower (all *P* < 0.0001). It was worth noting that 35.5% of CNALT patients had PLT < 150 000/μL. All fibrosis markers were significantly lower for CNALT patients (all *P* < 0.0001). There was no remarkable difference in SVR rates between the CNALT (97.8%) and non‐CNALT (95.3%) groups. The cumulative incidence of HCC after DAA treatment was comparable between the CNALT and non‐CNALT groups by Kaplan–Meier analysis (*P* = 0.117, log‐rank test) (Fig. 3a).

The cumulative incidence of HCC was significantly higher in non‐SVR patients than in SVR patients by Kaplan–Meier analysis (*P* < 0.0001). However, because prior HCC was the highest independent prediction factor in Kaplan–Meier testing (Fig. 3b), we divided the cohort into two groups, HCC history (+) and HCC history (−), for comparisons of clinical characteristics.

### *Comparisons of clinical characteristics between CNALT and non‐CNALT and HCC recurrence in the HCC history (+) group*

The clinical characteristics of patients with a history of HCC are shown in Table 2. We observed that 64.3% of patients had PLT < 150 000/μL. The SVR rate was 88.6%. Of the 70 patients, 23 (32.9%) had CNALT. In comparisons of clinical markers between CNALT and non‐CNALT groups, non‐CNALT patients had significantly lower scores for PLT (*P* = 0.030) and albumin (*P* = 0.046) but higher scores for liver fibrosis markers (*P* < 0.05, each) except autotaxin. Of 70 patients, 29 patients (41.4%) experienced HCC recurrence after DAAs during a median follow‐up period of 12.8 months. The cumulative recurrence rates of HCC were similar between the CNALT and non‐CNALT groups by Kaplan–Meier analysis (*P* = 0.979, log‐rank test) (Fig. 3c) and eventually reached 50% in both groups. The cumulative recurrence rate of HCC was also comparable among genotype 1 CNALT, genotype 1 non‐CNALT, genotype 2 CNALT, and genotype 2 non‐CNALT according to Kaplan–Meier analysis (all *P* > 0.05, log‐rank test).

**Table 2:** Clinical characteristics of patients with HCC history and comparisons of clinical features between CNALT and non‐CNALT groups

	Patients with HCC history (*n* = 70)	IQR	CNALT (*n* = 23)	Non‐CNALT (*n* = 48)	*P* value
Age at entry (years)	72	66–76	73	69	0.252
Male, *n* (%)	43 (61.4)		14 (60.9)	29 (61.7)	0.946
Genotype 1/2/other	57/13/0		18/5/0	39/8/0	0.634
Laboratory data					
Platelet count (/μL)	131 000	89 500–162 000	153 000	120 000	0.030
Platelet count <150 000/μL, %	64.3		47.8	73.9	0.032
Albumin (g/dL)	3.8	3.5–4.1	3.9	3.7	0.046
AST (U/L)	41	32–76	31	54	<0.0001
ALT (U/L)	39	27–66	24	53	ND
AFP (ng/mL)	6.3	4.0–17.2	4.0	11.5	0.0005
Fibrosis markers					
FIB‐4 index	3.97	2.77–6.31	3.07	5.50	0.005
APRI	0.85	0.50–1.84	0.48	1.47	<0.0001
M2BPGi (COI)	2.81	1.49–5.22	1.68	3.73	0.027
Autotaxin (mg/L)	1.64	1.49–2.48	1.48	1.80	0.171
DAA outcome of SVR, *n* (%)	62 (88.6)		20 (87.0)	42 (89.4)	0.766
Follow‐up period† (months)	12.8	5.93–31.7	13.5	12.3	0.586

† Interval between SVR12 and last outpatient clinic visit or HCC complication.

AFP, alpha fetoprotein; ALT, alanine aminotransferase; APRI, aspartate aminotransferase‐to‐platelet ratio index; AST, aspartate aminotransferase; CNALT, hepatitis C patients with normal alanine aminotransferase; COI, cutoff index; DAA, direct‐acting antiviral; FIB‐4, fibrosis‐4 index; HCC, hepatocellular carcinoma; IQR, interquartile range; M2BPGi, macrophage galactose‐specific lectin‐2 binding protein glycosylation isomer; ND, not determined; SVR, sustained virologic response.

#### *HCC recurrence factors in CNALT patients with the HCC history*

**Figure 4:**
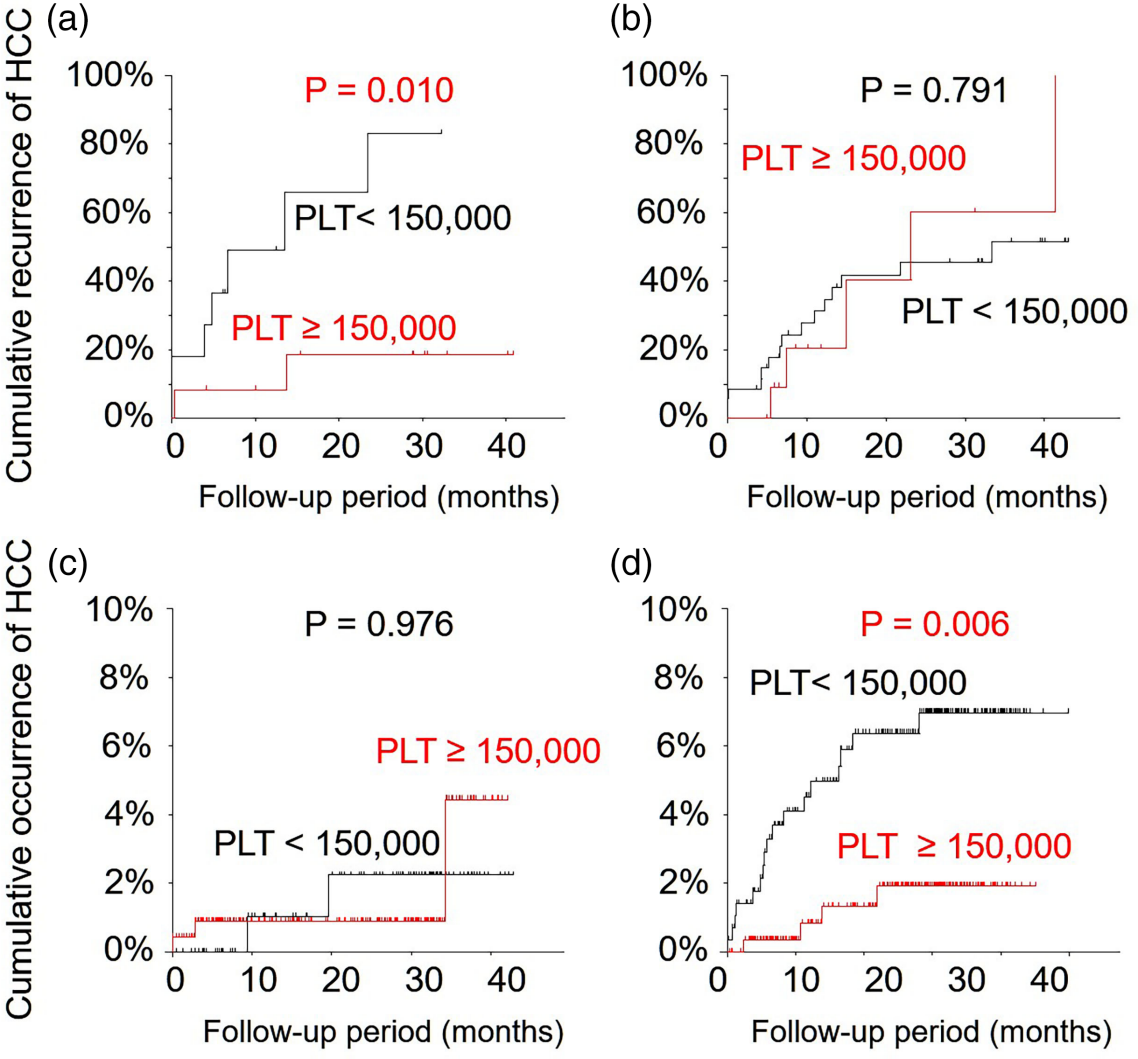
Cumulative incidence of HCC recurrence and HCC occurrence after DAA treatment in terms of PLT status between CNALT and non‐CNALT patients. The CNALT group with PLT ≥150 000/μL had a significantly lower cumulative incidence of HCC recurrence according to Kaplan–Meier testing (a). The non‐CNALT group showed no differences by Kaplan–Meier testing (b). While the CNALT group exhibited no remarkable differences by Kaplan–Meier testing (c), the non‐CNALT group with PLT < 150 000/μL had a significantly higher cumulative incidence of HCC occurrence according to Kaplan–Meier analysis (d). HCC, hepatocellular carcinoma; PLT, platelet count.

In a subanalysis of the CNALT group, the cumulative incidence of HCC recurrence was significantly lower for the PLT ≥150 000/μL group than for the PLT < 150 000/μL group by Kaplan–Meier analysis (*P* = 0.010, log‐rank test) (Fig. 4a). No other factors, such as albumin, AFP, fibrosis markers, or achievement of an SVR, were related with HCC recurrence. In addition, PLT ≥150 000/μL was significantly associated with a lower risk of HCC recurrence after adjustment by gender and age by Cox proportional regression analysis (odds ratio: 0.145, 95% confidence interval: 0.029–0.728, *P* = 0.019). However, this significance was absent in a subanalysis of the non‐CNALT group (Fig. 4b).

### *Comparisons of clinical characteristics between CNALT and non‐CNALT and HCC occurrence (de novo HCC) in the HCC history (−) group*

The clinical characteristics of patients without prior HCC history are presented in Table 3. A total of 44.0% of patients demonstrated PLT < 150 000/μL. Of 932 patients, 351 (37.7%) had CNALT status noted. The SVR rate was 98.0%. In comparisons of clinical markers, HCC history (−) CNALT patients had higher PLT (*P* < 0.0001) and albumin (*P* = 0.002) but lower liver fibrosis marker scores (all *P* < 0.0001). Of 932 patients, 26 patients (2.8%) experienced HCC occurrence after DAAs during a median follow‐up period of 26.1 months. There was no significant difference in the cumulative incidence of HCC occurrence between the CNALT and non‐CNALT groups by Kaplan–Meier analysis (*P* = 0.074, log‐rank test) (Fig. 3d). The cumulative occurrence rate of HCC was similar among genotype 1 CNALT, genotype 1 non‐CNALT, genotype 2 CNALT, and genotype 2 non‐CNALT according to Kaplan–Meier analysis (all *P* > 0.05, log‐rank test).

**Table 3:** Clinical characteristics of patients without HCC history and comparisons of clinical features between CNALT and non‐CNALT groups

	Patients without HCC history (*n* = 932)	IQR	CNALT (*n* = 351)	Non‐CNALT (*n* = 581)	*P* value
Age at entry (years)	69	60–75	69	69	0.404
Male, *n* (%)	384 (41.2)		144 (41.0)	240 (41.3)	0.932
Genotype 1/2/other	646/282/4		228/123/0	418/159/4	0.016
Laboratory data					
Platelet count (/μL)	157 000	115 000–197 000	174 000	150 000	<0.0001
Platelet count <150 000/μL, %	44.0		34.8	49.6	<0.0001
Albumin (g/dL)	4.1	3.9–4.3	4.2	4.1	0.002
AST (U/L)	37	27–57	25	50	<0.0001
ALT (U/L)	34	23–59	20	50	ND
AFP (ng/mL)	4.1	2.7–7.0	3.1	5.0	<0.0001
Fibrosis markers					
FIB‐4 index	2.91	1.87–4.56	2.28	3.38	<0.0001
APRI	0.62	0.36–1.12	0.36	0.88	<0.0001
M2BPGi (COI)	1.74	1.08–3.26	1.24	2.32	<0.0001
Autotaxin (mg/L)	1.483	1.09–2.02	1.25	1.62	<0.0001
DAA outcome of SVR, *n* (%)	913 (98.0)		347 (98.9)	566 (97.4)	0.131
Follow‐up period† (months)	26.1	9.3–32.6	24.0	26.5	0.044

† Interval between SVR12 and last outpatient clinic visit or HCC complication.

AFP, alpha fetoprotein; ALT, alanine aminotransferase; APRI, aspartate aminotransferase‐to‐platelet ratio index; AST, aspartate aminotransferase; CNALT, hepatitis C patients with normal alanine aminotransferase; COI, cutoff index; DAA, direct‐acting antiviral; FIB‐4, fibrosis‐4 index; HCC, hepatocellular carcinoma; IQR, interquartile range; M2BPGi, macrophage galactose‐specific lectin‐2 binding protein glycosylation isomer; ND, not determined; SVR, sustained virologic response.

#### *HCC occurrence factors in non‐CNALT patients without HCC history*

In a subanalysis of the non‐CNALT group, the cumulative incidence of HCC occurrence was significantly higher for the PLT < 150 000/μL group than for the PLT ≥150 000/μL group by Kaplan–Meier analysis (*P* = 0.006, log‐rank test) (Fig. 4d). Several factors were also associated with HCC occurrence, including age, male gender, albumin, AFP, and fibrosis markers (all *P* < 0.05). PLT < 150 000 /μL was a marginal predictor of HCC occurrence in Cox proportional regression analysis (odds ratio: 3.80, 95% confidence interval: 0.85–17.00, *P* = 0.081), while male gender (odds ratio: 3.43, 95% confidence interval: 1.20–9.83, *P* = 0.021) and albumin <4.0 g/dL (odds ratio: 7.98, 95% confidence interval: 1.76–36.24, *P* = 0.007) were demonstrated to be independent risk factors of HCC occurrence. No such differences were found in a subanalysis of the CNALT group (Fig. 4c).

## Discussion

This modern DAA‐era study supports previous IFN‐based reports on normal ALT patients having a milder degree of liver fibrosis than abnormal ALT patients.^8^ The benefits of HCV treatment before the progression to hepatic disease include several‐fold decreases in the risk of death and development of HCC.^18^ It was recently found that microbiota dysbiosis in the gut was associated with disease progression in HCV patients and was already evident in the normal ALT stage,^19^ supporting a commencement of DAAs before signs of disease advancement. Whether microbiota dysbiosis in CNALT patients is reversible after HCV eradication requires future study.

This investigation also demonstrated that SVR rates for CNALT and non‐CNALT cases were comparable in the DAA era, as was reported for IFN.^20^ Accordingly, CNALT patients may be considered candidates for DAA treatment toward eradicating HCV worldwide. Due to the high cost of DAAs, however, the health economics of HCV eradication in terms of nonprogression of liver status and subsequent HCC reduction need further consideration.

Finally, previous affliction was the strongest predictor of HCC development after DAAs in this cohort as reported elsewhere.^5^ This observation led us to consider two additional subgroups: HCC past history (+) and HCC past history (−). We observed that HCC recurrence was lower for CNALT + PLT ≥150 000/μL, while HCC occurrence was higher for non‐CNALT + PLT < 150 000/μL. An HCC history (+) group was reportedly associated with significant progression of liver fibrosis compared with a HCC (−) group.^21^ Regarding the disease progression status of the HCC history (+) group, normal ALT might have been related to advanced liver damage by hepatocyte loss, as in liver cirrhosis. Therefore, lower PLT + normal ALT could indicate higher HCC recurrence. On the other hand, in the HCC history (−) group, patients showed a milder degree of liver fibrosis as in chronic hepatitis, whereby lower PLT + higher ALT (i.e. more active hepatitis) could be associated with HCC occurrence. Indeed, a previous study evidenced that relatively high ALT and low PLT were closely associated with HCC development in HCV patients with normal ALT levels,^22^ which supported our results in terms of HCC occurrence. Clinicians should pay careful attention to such cases during post‐DAA surveillance. Extra vigilance is also required for patients with prior HCC as such cases exhibited recurrence at a 50% rate.

There were three major limitations in this study. First, the ALT levels of HCV patients fluctuate over time. As such, the clinical effects of ‘persistently’ normal ALT of more than 1 year as defined previously^8^ should be strictly analyzed in the future. Second, the HCC surveillance interval duration differed for each case and depended on the discretion of the attending physician. Thus, we cannot exclude the possibility that HCC surveillance interval duration may influence HCC recurrence or occurrence detection; this issue requires assessment in a future study. Third, the need for longer external validation in a larger cohort is warranted due to the small number of HCC cases after DAAs in this investigation.

In conclusion, CNALT patients exhibit a milder degree of liver fibrosis in the DAA era but may be considered for DAA treatment in consideration of the high SVR rate toward the eradication of HCV worldwide. The combination of CNALT + PLT and non‐CNALT + PLT might be useful for HCC recurrence and HCC occurrence surveillance after DAAs in the clinical setting.
